# Cardiovascular disease (CVD) risk assessment of HIV medication regimens using hematopoietic CD34+ progenitor cells

**DOI:** 10.1186/s13287-022-02775-6

**Published:** 2022-03-07

**Authors:** Adrian Farid Elzarki, Seshagiri Rao Nandula, Hassan Awal, Gary L. Simon, Sabyasachi Sen

**Affiliations:** 1 Department of Medicine (Endocrinology) and Biochemistry and Molecular Medicine, George Washington University School of Medicine and Health Sciences, Washington, DC 20037 USA; 2 Veterans Affairs Medical Center, Washington, DC 20422 USA

**Keywords:** HIV, CVD, Non-nucleoside reverse transcriptase inhibitors, Integrase inhibitor

## Abstract

**Background:**

To determine the effects of integrase inhibitor (INSTI) in comparison with non-INSTI-based regimens such as non-nucleoside reverse transcriptase inhibitors (NNRTIs)-based regimens on cardiovascular disease (CVD) risk in HIV+ patients without overt history of CVD or diabetes, with normal CD4:CD8 count. For CVD risk assessment we primarily used hematopoietic CD34+ progenitor cells, as a biomarker.

**Methods:**

Nineteen male subjects, ages 32–61 years with BMI 21.0–36.0, were enrolled. This was a single time point, cross-sectional, observational study. Subjects were enrolled under 2 groups (either on INSTI-based regimen with 13 subjects or NNRTI (non-INSTI)-based regimens with 6 subjects) who were taking stable doses of HAART. The medication regimens were a combination of one NRTI (typically tenofovir–emtricitabine) plus one INSTI or NNRTI. Our outcome measures were focused on cardiovascular and endothelial cell function and systemic inflammation. Our primary outcome measures were peripheral blood-derived hematopoietic progenitor cell number (CD34 and CD133 positive), CD34+ cell function and gene expression studies. Our secondary outcomes were arterial stiffness measures and serum-based markers of inflammation.

**Results:**

A significant increase in percentage number of progenitor cells, CD133+ cells (*p* = 0.004), was noted along with an increase of double progenitor mark positive CD133+/CD34+ progenitor cell population being observed in INSTI group as compared to NNRTI group, by flow cytometry. mRNA gene expression for antioxidant gene catalase was noted along with a trend toward a decrease in gene expression of inflammatory marker IL6 (*p* = 0.06) being observed in CD34+ from INSTI group vs NNRTI group. The plasma IL-6 and CRP levels did not change significantly between the groups. Neutrophil–Lymphocyte ratio (NLR), an important marker of inflammation, was noted to be lower in INSTI group. A mean fasting glucose level was also lower in the INSTI group compared to NNRTI group (*p* = 0.03). Interestingly, urine microalbumin levels were higher in the INSTI group compared to NNRTI group (*p* = 0.08), while eGFR levels were significantly lower in the INSTI group (*p* = 0.002). The arterial stiffness measures did not show statistically significant differences between the two groups.

**Conclusion:**

We conclude that the INSTI regimen may provide a better CVD risk profile compared to NNRTI-based HAART regimen; however, the increased albuminuria along with lower eGFR, noted in INSTI group, is of concern. Because of the small size, these results would need replication in additional studies before changing clinical practice.

*Clinical trial registration* https://clinicaltrials.gov/ct2/show/NCT03782142?cond=Hiv&spons=Sabyasachi+sen&cntry=US&state=US%3ADC&city=Washington&draw=2&rank=1. ClinicalTrials.gov Identifier: NCT03782142.

## Introduction

Human immunodeficiency virus (HIV) is a retrovirus belonging to the lentivirus genus that attacks the human immune system over long periods of time [1]. The prevalence of HIV has been described as a global epidemic [2]. According to the CDC, in 2018 nearly 38,000 new cases in the USA and 1.7 million new cases worldwide were reported. The total number of reported individuals with HIV reached approximately 38 million globally. Meanwhile, an estimated 770,000 individuals died as a result of HIV-AIDS related illness that same year [3].

Cardiovascular diseases (CVD) are one of the leading causes of morbidity and mortality in antiretroviral-treated people living with HIV (PWH) with risk score algorithms based on traditional risk factors being shown to be consistently unreliable in estimating risk in this population. Although persistent associations with inflammatory markers and CVD have been demonstrated, few biomarkers have emerged as being clinically useful. [4–6]. Cell-based assays in the realm of HIV and CVD risk assessment are almost nonexistent although the presence of endothelial cell dysfunction in HIV has been well established [7].

There are number of major classes of antiretroviral therapy (ART) which are currently in use. Although their toxicity profile has been investigated [8], effects on long-term CVD risk in HIV remains largely unexplored. The three major categories of medication are as follows: protease inhibitors (PI), integrase strand inhibitors (INSTI) and nucleoside and (NRTIs) and non-nucleoside reverse transcriptase inhibitors (NNRTIs) [9].

Currently, most patients are treated with combination antiretroviral therapy initiated at the time of diagnosis. Combination therapy is utilized to reduce the risk of mutational resistance [10]*.* Currently, the most frequently employed regimens involve a combination of NRTIs, NNRTIs and/or integrase inhibitors (INSTIs).

HIV infection and some of the antiretroviral (ART) regimens have been associated with adipose tissue/lipodystrophy changes and disorders of glucose and lipid metabolism. Metabolic and endocrine perturbations including insulin resistance, diabetes and dyslipidemia have been of significant concern in human immunodeficiency virus (HIV)-infected individuals [11, 12].

HIV-infected individuals may be at risk of accelerated atherosclerotic cardiovascular disease (CVD) and metabolic syndrome. More recent data suggest that immune activation and inflammation from chronic HIV infection may also play an important role in HIV-associated metabolic dysfunction [13].

The long-term effects of ART on the cardiovascular system, particularly the newer generation regimens containing INSTI or NNRTI’s effect on endothelial cell function, hematopoietic stem cell function and cardiovascular system in general, have not been investigated.

Although the etiology of metabolic/endocrine complications in HIV-infected individuals despite viremic control is poorly understood, it is most likely related to the interplay of host, viral and ART factors and the complex interactions among the long-term consequences of infection, chronic ART and the underlying inflammatory process. As noted above it is important to determine which ART regimens promote or reduce the chances of endothelial and cardiometabolic dysregulation, particularly chronically.

We hypothesize that HIV regimens will lead to differences in the degree of cardiometabolic derangement and stem cell number, function and gene expression. *Our goal was to establish or refute the cardiovascular benefits of using an INSTI-based HAART regimen using hematopoietic cell (CD34* +*)-based results.*

## Methods

In our study we decided to use adult stem cells such as hematopoietic progenitor cells (CD34 +) as our primary outcome measure [14]. Our laboratory group has used hematopoietic cells as a cell-based diagnostic and prognostic biomarker in multiple scenarios and have evaluated ECD evaluation modalities such as arterial stiffness measures [15], flow mediated dilatation and podocyte inflammatory markers [16–19].

This is a cross-sectional observational study examining patients with HIV and a history of taking either INSTI or NNRTI regimens for at least one year. The study was conducted in accordance with Good Clinical Practice guidelines set forth by the International Conference of Harmonization as well as local regulatory guidelines with the approval and oversight of the George Washington University Institutional Review Board.

Subjects were initially pre-screened to assess eligibility. Once determined preliminary eligibility, they were brought in for a screening visit to confirm eligibility via interview, medical record check and laboratory workup once the subject signed the informed consent form.

The study entailed a single time point visit. The assessments that were completed include: vital measurements such as BMI, blood pressure, heart rate, adverse event (AE) checks and a peripheral blood draw. Approximately 60 ml of blood was drawn for CD34+ endothelial progenitor cell harvesting and routine blood work. Other parameters tested were measurement of waist-to-hip ratio, Tanita body composition scale, pulse wave analysis and pulse wave velocity to determine arterial stiffness.

A follow-up phone call visit was completed 30 days from the last in-person visit to assess for any residual adverse events (AE).

### Participants

The subjects were included if they were males between 40 and 70 years old. Patients with BMI between 25 and 39.9 kg/m^2^ were included. We avoided subjects with severe obesity (BMI ≥ 40) as those subjects are deemed to have pre-existing CVD risk). Patients with normal and mildly impaired renal function were included, with lowest eGFR cutoff of 50 ml/min/1.73 (GFR, as calculated by MDRD formula).

Any patients with uncontrolled hyperglycemia, history of liver disease, clinically significant RBC cell disorders, HBV or HCV, chronic malabsorption, statin medication use, use of consistent steroid medications and untreated thyroid disease were excluded. Additional Inclusion and Exclusion criteria can be found in Appendix.

### Outcome objectives

The objective is to detect differences between INSTI- and NNRTI-based HAART-based regimens Our primary outcome measures were cellular outcome measures pertaining to the cardiovascular–hematopoietic system with secondary outcome measures being measures of vascular function such as arterial stiffness [measured by pulse wave analysis (Augmentation Index) and pulse wave velocity (m/s)], and serum biochemistry analysis pertaining to cardiometabolic function parameters: such as hemoglobin A1C(HbA1C) (as a reflection of glycemic control), fasting lipid profile, C-reactive protein (inflammation), adiponectin (measure of endothelial paracrine function), fasting Insulin levels (measure of insulin resistance) and interleukin-6 (inflammation).

The cell-based measures evaluating the function of CD34+ cells were to ascertain the effects of NNRTI- and INSTI-based regimens on CD34+ cell number, (CD34+ cell number percentage, (%CD34+ve out of total mononuclear cells, MNC, population), CD34+ cell function (cell migration function in response to a chemotactic agent, stromal-derived factor-1a, SDF1α) and CD34+ cell gene expression (specifically genes related to endothelial function) in patients with HIV.

#### Secondary outcome measures

##### Body composition measurement

Body composition was measured manually as well as using Tanita™ BF-350 Body Composition Scale (an impedance scale). Manual measurement included height, waist circumference, hip circumference and BMI calculation. Tanita scale (Tanita Corporation of America, Inc, USA) uses a bio-impedance electrical impulse to measure body fat percent, fat mass (kg), fat-free mass (kg), percent body water and water mass (kg) alongside weight. It also calculates the subject’s BMI and estimated basal metabolic rate (BMR).

##### Arterial stiffness

This parameter was measured using Atcor Sphygmocor CP system (Atcor Technologies, USA). We obtained two outcomes: pulse wave velocity and pulse wave analysis.

Pulse wave analysis (PWA) was measured on the left radial artery with the subject supine. At least three readings were taken with Operator Index ≥ 80. Measurement includes augmentation index (AI), augmentation index adjusted for heart rate of 75 (AI-75), augmentation pressure (AP), aortic and radial reading of systolic, diastolic, pulse pressure and mean pressure.

Pulse wave velocity (PWV) was measured with the subject supine. This measurement requires a distal and proximal artery point delineation. Right femoral artery was used as the distal point with proximal being the left carotid. A straight line if drawn between these two points would include the heart. Index and ring fingers were used to manually localize the pulse, sometimes an arterial Doppler was used to localize the femoral pulse on patient with challenging body habitus. Once a stable pulse waveform was observed, the probe position was kept stable for 20 more pulses before the reading was finalized. Three readings were taken with standard deviation of less than 10%. The result reported a velocity in m/s, with standard error of the mean.

### Biological sample and vital statistics collection

A venous blood sample was collected from the antecubital fossa. About 60 mL of blood was collected, centrifuged at 4 °C and plasma stored at − 80 °C until analysis or sent immediately to LabCorp. 60 ml for EPC analysis and 20 ml for standard of care blood tests included basic metabolic panel, lipid panel, HbA1c, fasting glucose, hsCRP, IL6, adiponectin and insulin. Urine sample was collected for urine microalbumin and creatinine ratio. Vital statistics were measured on the left arm including systolic pressure, diastolic pressure and heart rate, and long sublingual temperature.

### Cellular and clinical assessments

#### *CD34+endothelial progenitor cell analysis*

Peripheral blood samples (approximately 60 ml) were drawn from patients and phosphate buffered saline (1:1) was added. Identification and quantification of circulating cell phenotypes was performed on fresh blood samples, within 3 h after collection, using flow cytometry. Briefly, mononuclear cells (MNCs) were then isolated from whole blood using a Ficoll density centrifuge method. MNCs were counted and an aliquot was used for CFU-Hill colony formation assay following the manufacturer’s instruction (Stem Cell Technologies, Vancouver, BC, Canada). Colony forming units were counted at day 14. A fraction of the MNC were stained with fluorescein isothiocyanate (FITC)-conjugated antihuman CD34, Allophycocyanin (APC) conjugated antihuman CD184 (CXCR4), Allophycocyanin (APC) conjugated antihuman CD133 and FITC conjugated antihuman CD31 antibodies (Miltenyi Biotec GmbH, Bergisch-Gladback, Germany) in order to analyze specific progenitor cell surface markers (CD34 and CD133) and mature endothelial cell surface markers (CD31) or receptors for SDF1a ligand, CXCR4) by flow cytometry. After gating mononuclear cells in the side scatter (SSC)-A vs forward scatter (FSC)-A plot, CD34/CD33/CD184 single- and double-positive cells were identified. Cells were acquired on a fluorescence-activated cell sorter (FACS) Canto instrument (Becton Dickinson, USA) and scored with the Flo-Jo software.

To isolate EPCs (CD34+), MNCs were magnetically sorted through a column after cells were stained with CD34+ve microbeads antibody (Miltenyi Biotec GmbH, Bergisch Gladback, Germany). An aliquot of CD34+ cells were then stained with trypan blue and counted using an Auto Cellometer Mini (Nexcelom Bioscience, USA) to assess viability.

CD34+ gene expression analysis was performed by quantitative reverse transcriptase polymerase chain reaction (qRT-PCR) as previously described (16). CD34+ cell total mRNA was extracted and purified using the RNeasy Minikit (Qiagen, Germany). mRNA was then converted into cDNA by using the high-capacity cDNA reverse transcriptase kit (Thermo Fisher Scientific, MA). Possible gene expression changes were assessed by a CFX96 real-time PCR systems (Bio-Rad, CA.) using Taqman Universal masters Mix II (Thermo Fisher Scientific, USA) and inventoried probes. The gene expression analysis included antioxidants, apoptosis, endothelial functions, chemotaxis, inflammation and endothelial lineage cell surface markers. The expression of each individual gene was normalized to either housekeeping 18S or mean Cq values are reported.

The migratory capacity of CD34+ was evaluated using the CytoSelect 24-well Cell Migration Assay kit (Cell Biolads, Inc., San Diego, CA). Cells were suspended in serum-free media and seeded at 100,000 cells per insert. Migration of the cells through a 3-um polycarbonate membrane to the wells containing a serum-free media (control) and chemoattractant SDF-1α (10 or 100 ng/mL) (from Sigma-Aldrich, USA) was assessed after cells were kept overnight in incubator. Migratory cells were dissociated from the membrane and subsequently lysed and quantified by fluorescence (480 nm/530 nm) using CyQuant GR dye (Cells Biolabs, Inc, USA). The fluorescence ratios between cells exposed to the chemotactic factor and cells exposed to chemoattractant-free media (control) along the visits were used to analyze the migratory capacity of the cells.

### Statistical methods

Because of the small sample size in this pilot program study, the analysis emphasis is on descriptive statistics. Differences between the INSTI and NNRTI groups were compared by Mann–Whitney tests. Significance test results indicate rough markers of parameters which may be affected by treatment regimen and not as confirmation of treatment effects. To balance Type 1 and 2 error rates for this preliminary study, no adjustments were made for multiple testing.

## Results

### Study sample

A total of 19 subjects were enrolled into two different groups taking two different classes of Antiretroviral regimen, either INSTI-based regimen (13 subjects) or NNRTI-based regimen (6 subjects). After several months of recruitment attempts, it was evident that recruiting more than a handful of patients in the protease inhibitors group would not be successful. We therefore dropped that group. We were able to attain a final sample size of *n* = 13 for the INSTI group and *n* = 6 patients in the NNRTI group.

Characteristics of the 2 groups of patients are shown in Table 1. Outcome differences are in Table 2. Table 3 shows the prominent components of the HAART regimen for each subject included in either of the two groups.

**Table 1:** Baseline characteristics between the two groups

Parameter	INSTI (*n* = 13)	NNRTI (*n* = 6)	Mann–Whitney *p* value
Age	49.46 (33–61)	44.5 (32–59)	
Sex (male only)	13	6	
African American	11	3	
Caucasian	2	3	
Systolic	135.30 ± 7.53	132.33 ± 8.17	0.9488
Diastolic	83.76 ± 3.76	80 ± 4.47	0.8483
Pulse	74.46 ± 3.51	68.66 ± 6.07	0.2916
Temp	97.98 ± 0.13	98.48 ± 0.11	0.0283
BMI	27.42 ± 1.28	26.74 ± 1.99	0.8025
Weigh Kgs	82.04 ± 3.88	76.36 ± 9.80	0.7166
Waist in CM	95.08 ± 3.67	93.4 ± 4.84	0.8995
Hip in CM	103.25 ± 2.98	102.6 ± 3.98	0.9360

**Table 2:** All results between two groups

Parameters	INSTI (*n* = 13)	NNRTI (*n* = 6)	Mann–Whitney *p* value
Avg PWV	8.72 ± 0.50	7.62 ± 0.78	0.1998
Avg Aug Index_75	17.08 ± 3.97	12.83 ± 4.85	0.3974
Avg Aug Index	21 ± 3.86	18.16 ± 3.96	0.2587
Avg Aug pressure	9.08 ± 1.85	7.83 ± 2.85	0.4202
Glucose	91.58 ± 3.71	100.66 ± 2.78	0.0379
Uric acid	5.75 ± 0.35	5.61 ± 0.76	0.8379
BUN	15.45 ± 1.60	13.66 ± 1.68	0.6454
Creatinine (serum)	1.15 ± 0.05	0.92 ± 0.05	0.2838
Sodium	140.33 ± 0.89	140.33 ± 0.91	0.8378
Potassium	4.30 ± 0.12	4.2 ± 0.11	0.9149
Chloride	103 ± 0.93	104 ± 0.68	0.7990
AST (SGOT)	41.25 ± 16.53	28 ± 6.63	0.8685
ALT (SGPT)	43 ± 20.14	30 ± 7.18	0.4227
Cholesterol	178.16 ± 14.65	203.66 ± 11.55	0.4240
Triglycerides	139 ± 28.99	128.83 ± 19.97	0.6317
HDL cholesterol	53.25 ± 4.18	53 ± 6.02	0.8729
VLDL cholesterol	27 ± 5.70	24 ± 3.44	0.6655
LDL cholesterol	97.91 ± 11.25	126.66 ± 12.52	0.1306
LDL/HDL	1.94 ± 0.24	2.6 ± 0.46	0.2496
URINE-creatinine	132.76 ± 18.78	139.3 ± 35.69	0.99
URINE microalbumin	102.92 ± 68.05	10.75 ± 4.45	0.0851
URINE microalb/creat ratio	66.76 ± 36.71	8.46 ± 2.53	0.2091
eGFR	59 ± 8.04	106.16 ± 4.94	0.0022
Serum CRP	1.73 ± 0.37	2.27 ± 0.69	0.4936
Serum IL6	3.04 ± 0.57	1.72 ± 0.45	0.6250
Total WBC	5.54 ± 0.44	5.66 ± 1.29	0.6657
Total RBC	4.50 ± 0.13	4.95 ± 0.30	0.2496
Hemoglobin	13.59 ± 0.45	14.63 ± 0.35	0.1856
Hematocrit	40.90 ± 1.08	42.81 ± 1.36	0.4792
MCV	90 ± 1.41	86.83 ± 2.61	0.2251
MCH	30.26 ± 0.83	29.95 ± 1.34	0.8375
MCHC	33.15 ± 0.48	34.36 ± 0.60	0.0973
RDW	13.91 ± 0.24	13.41 ± 0.72	0.2793
Platelet	230 ± 15.87	204.83 ± 22.58	0.2500
Neutrophil	46.66 ± 3.82	58 ± 2.19	0.0870
Lymphocyte	40.25 ± 3.12	32 ± 1.89	0.1299
Monocyte	9.16 ± 0.66	7.66 ± 0.55	0.1648
HbA1c	5.32 ± 0.13	5.18 ± 0.20	0.6016
Leptin	13.51 ± 3.69	8.43 ± 2.33	0.6165
Adiponectin	4.58 ± 0.81	5.06 ± 1.26	0.8262
Insulin	11.66 ± 2.14	18.86 ± 10.08	0.9501
MNC	1.10E+08 ± 1.44E+07	1.01E+08 ± 1.89E+07	0.7815
%CD34+	1.57 ± 0.05	1.33 ± 0.54	0.9157
SDF10/control	0.83 ± 0.13	1.21 ± 0.25	0.2786
CFU Day 14	11.73 ± 3.86	16.37 ± 3.10	0.1697
*CD34+* *cell gene expressions*			
CAT	33.50 ± 1.13	30.48 ± 0.64	0.1320
SOD2	33.12 ± 1.62	29.17 ± 0.71	0.5728
VEGFA	34.50 ± 0.95	36.29 ± 0.78	0.4762
PECAM1	34.43 ± 1.42	32.01 ± 0.68	0.8182
EDN1	36.01 ± 0.18	37.1 ± 0.62	0.2333
NOS3	39.4 ± 0.27	39.11 ± 0.73	0.7619
CDKN1A	32.56 ± 1.22	32.39 ± 0.82	0.7922
TP53	33.37 ± 0.89	31.68 ± 0.76	0.2662
IL6	36.65 ± 0.63	38.81 ± 0.36	0.0635
TNF	34.10 ± 0.60	35.32 ± 0.82	0.4762
GPX3	38.82 ± 1.03	38.27 ± 0.88	0.9307
CXCL12	38.97 ± 0.26	37.71 ± 0.30	0.2286
CXCR4	33.56 ± 1.41	31.89 ± 0.86	0.6991
*Flow cytometry*			
% of CD34-FITC	4.67 ± 143	3.68 ± 1.06	0.9451
% of CD184-APC	74.6 ± 2.77	55.9 ± 9.25	0.1059
% of CD31-FITC	84.23 ± 8.17	43.84 ± 15.70	0.0539
% of CD133 APC	8.83 ± 2.23	0.25 ± 0.15	0.0040
% of CD34FITC+ CD184+ APC	11.78 ± 5.59	2.95 ± 0.69	0.5395
% of CD31FITC+ CD184APC+	63.13 ± 6.89	40.30 ± 11.50	0.3736
% of CD34 FITC+ CD133 APC	2.0 ± 0.96	0.29 ± 0.15	0.0759

Results that are significant or close to significance (at or below *p* = 0.05), are marked in Red. Results that are greater than *p* = 0.05 but less then *p* = 0.2 are marked in green

**Table 3:** Medications and doses of all 21 subjects enrolled in the study

Patient	Medication group	Medication name	Dose/unit	Freq
1	INSTI	Descovy + Isentress	600 (2)-200-25	QD
2	INSTI	Truvada and Tivicay	200	QD
3	INSTI	Triumeq	600-50-300	QD
4	INSTI	Triumeq	600-50-300	QD
5	INSTI	Genvoya	150-150-200	QD
6	INSTI	Biktarvy	50-200-25	QD
7	INSTI	Tivicay and Descovy	50-200-25	QD
8				
9	INSTI	Juluca	50-25	QD
10	NNRTI	Odefsey (Rilpivirine)	200-25-25	QD
11	INSTI	Juluca	50-25	QD
12	INSTI	Genvoya	150-150-200	QD
13	NNRTI	Odefsey	200-25-25	QD
14	NNRTI	Atripla (Efivarenz)	600-200-300	QD
15		*Patient dropped out*		
16	NNRTI	Odefsey	200-25-25	QD
17	INSTI	Biktarvy	50-200-25	QD
18	NNRTI	Odefsey	200-25-25	QD
19	NNRTI	Epzicom + Viramune	600-300	QD
20				
21	INSTI	Descovy + Isentress	200-25	QD
22	INSTI	Descovy + Tivacay	200-25	QD

### Cellular outcomes

#### Endothelial progenitor cell (CD34+) based assays

*Total number of MNCs* (1.10 × 10^8^ for INSTI group and 1.01 × 10^8^ for NNRTI group) and *the % of endothelial progenitor cells (CD34* +*)* purified from MNCs population were slightly higher in INSTI group (1.57 ± 0.05) as compared to NNRTI group (1.33 ± 0.54), although not statistically significant.

Colony forming units (CFU Assay): Results suggest higher CFU values in the NNRTI group (11.73 ± 3.86 in INSTI group and 16.37 ± 3.10 in NNRTI group) but not statistically significant, *p* = 0.16).

The migratory response of CD34+ cells to the chemotactic factor SDF1α (concentration of 10 ng/ml), an important functional property and assay of progenitor cells, was higher in INSTI group (0.83 ± 0.13) as compared to NNRTI group (1.21 ± 0.25), even though statistically not significant. Lesser distance from chemotactic factor target indicates better cell migratory property.

**Fig. 1:**
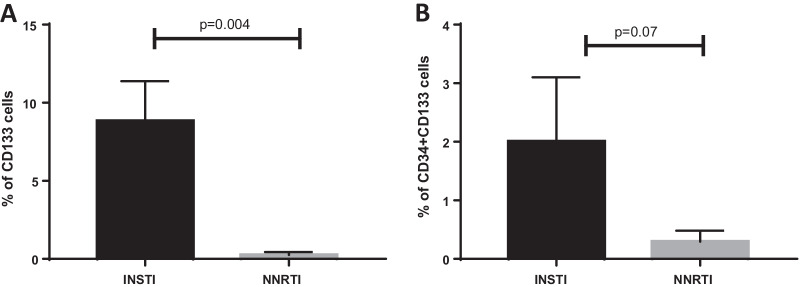
Cell based changes: a significant increase in percentage of CD133 positive hematopoietic progenitor cells (**a**, *p* = 0.004). A trend in increased dual positive CD34+ and CD133+ cells, close to statistical significance (**b**; *p* = 0.07) is observed in INSTI (*n* = 13) group as compared to NNRTI (*n* = 6) group

CD133+ progenitor cells (Fig. 1a) had a higher level in INSTI group (*p* = 0.004) as compared to NNRTI group. CD34+ CD133 (dual progenitor cell marker positive, Fig. 1b) cell number was also higher in the INSTI group with values approaching statistical significance (*p* = 0.07). CD34+ progenitor cell number (single progenitor cell marker positive) was also higher in the INSTI group.

### Gene expression analysis

**Fig. 2:**
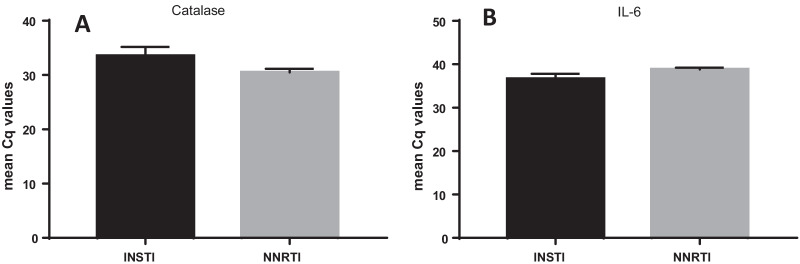
The effect of INSTI and NNRTI on CD34+ve cell mRNA gene expressions: the important antioxidant gene Catalase (**a**) expression increased (*p* = *0.13*) in INSTI (*n* = 13) group as compared to NNRTI (*n* = 6) group (**a**, *p* = 0.13). Concurrently, the inflammatory marker IL6 gene expression was lower (*p* = 0.06) in INSTI (*n* = 13) group as compared to NNRTI (*n* = 6) group, (**b**, *p* = *0.06*). However, the results did not reach statistical significance

CD34+ cell function represents vascular endothelial health, so we investigated the gene expression on CD34+ cells for antioxidants such as cytosolic Catalase, mitochondrial SOD2 (superoxide dismutase 2) and extra-cellular antioxidant GPX3 (glutathione peroxidase 3), of which catalase is the most investigated and an important one. The mean Cq values of cytoplasmic antioxidant, catalase was slightly higher (*p* = 0.13) in the INSTI group (Fig. 2a) and a trend in increased mean Cq values for SOD2, a mitochondrial antioxidant, (*p* = 0.57) was also observed in INSTI group compared to NNRTI group. The endothelial marker gene expressions, PECAM1 (*p* = 0.81) and VEGFA (*p* = 0.47) Cq, were nearly at the same level for the 2 groups. However, there was a decreased mean Cq value of the inflammatory marker Il-6 (*p* = 0.06) noted in the INSTI group (Fig. 2b), and no difference is seen in TNF-α value (*p* = 0.47) in INSTI group as compared to NNRTI group. Therefore, taking all hematopoietic stem cell-based results together it appears INSTI does provide some degree of vascular endothelial protection compared to NNRTI-based HAART regimens.

### Clinical outcomes

**Fig. 3:**
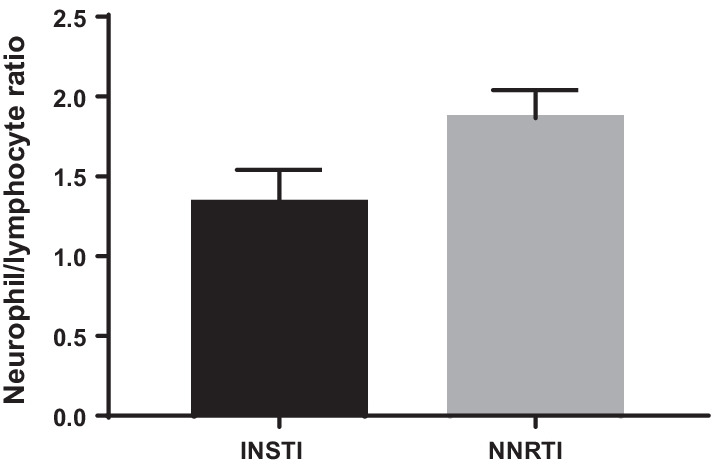
Neutrophil and lymphocyte ratio (NLR): a non-statistically significant decrease in NLR (*p* = 0.11) was observed in INSTI (*n* = 13) group as compared to NNRTI (*n* = 6) group. A decrease in NLR is associated with reduced inflammation

Table 2 shows blood biochemistry measures collected during the single time visit across the entire study group. Most of the parameters were not statistically significant between groups but were showing a trend toward lower systolic and diastolic pressure, BMI and higher Insulin levels in NNRTI group (Fig. 3).

### Venous blood biochemistries

Venous blood biochemistries were analyzed by LabCorp of America. We found no statistically significant differences in **Adipokines** such as leptin and adiponectin levels in NNRTI group as compared to INSTI group. Please note adiponectin particularly has been associated with improved vascular function (See Table 2, Results).

#### Serum markers of insulin sensitivity

**Fig. 4:**
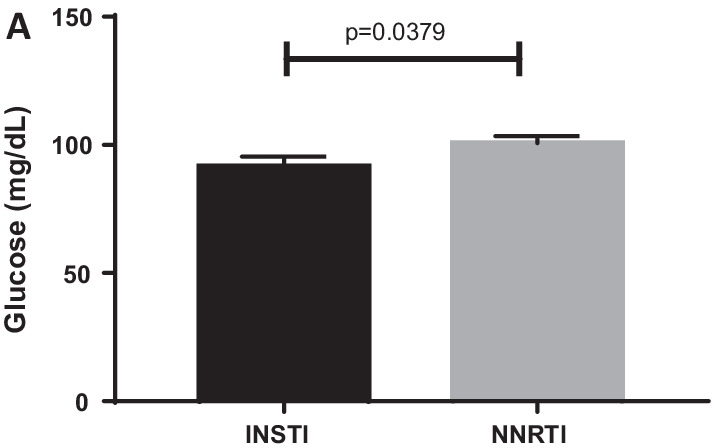
Effect on blood glucose: fasting glucose levels (**a**) were found to be lower in INSTI group, with statistically significant difference at *p* = 0.037. There was no difference in concomitant insulin values

Insulin levels demonstrate no evidence of a statistical difference (*p* = 0.95) between the two arms. In the NNRTI arm the insulin level was 18.86 (SEM + 10.08) while the INSTI arm was 11.66 (SEM + 2.14). Although there is no evidence of differences in insulin levels, it is interesting that the mean fasting glucose levels in the NNRTI group are significantly increased (*p* = 0.03) as compared to INSTI group (Fig. 4).

#### Inflammatory markers

**Fig. 5:**
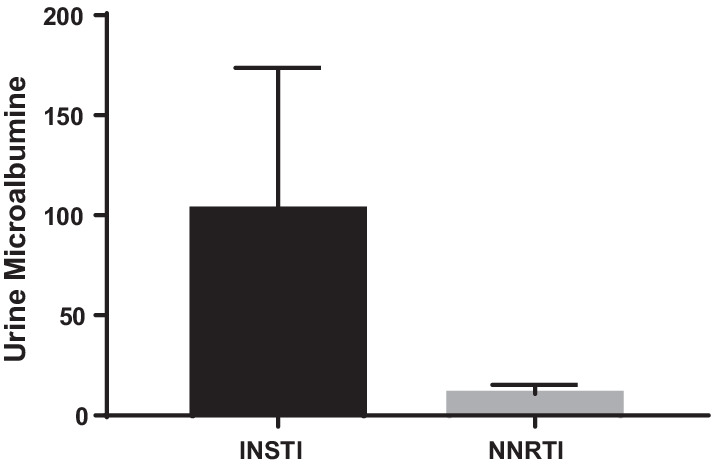
Urine microalbumin: interestingly, urine microalbumin levels were noted to be higher in INSTI (*n* = 13) group as compared to NNRTI (*n* = 6) group. The result was close to statistical significance (*p* = 0.08)

We analyzed lipid profile and inflammatory markers such as IL6 and CRP. Interestingly, IL6 showed a mean value in NNRTI arm of 1.72 (SEM + 0.45) and in the INSTI level arm was 3.04 (SEM + 0.57), although not statistically significant. CRP, also known as highly sensitive C-reactive protein, is an inflammatory marker which was compared between the two groups (Fig. 5b). In NNRTI group the mean value was higher at 2.27(SEM + 0.69) and in the INSTI group, there was a mean of 1.73 (SEM + 0.37), with no statistical difference.

#### Urine microalbumin

There was a nonsignificant trend for lower albuminuria levels in the NNRTI group (10.75 SEM + 4.45) compared to the INSTI group (102.92 SEM + 68.05, Fig. 5). For reference, normal albumin levels should be 30 mg/g or below, with elevated levels implying worsening kidney function. Though nonsignificant the *p* value was close to significance at 0.08.

#### eGFR

**Fig. 6:**
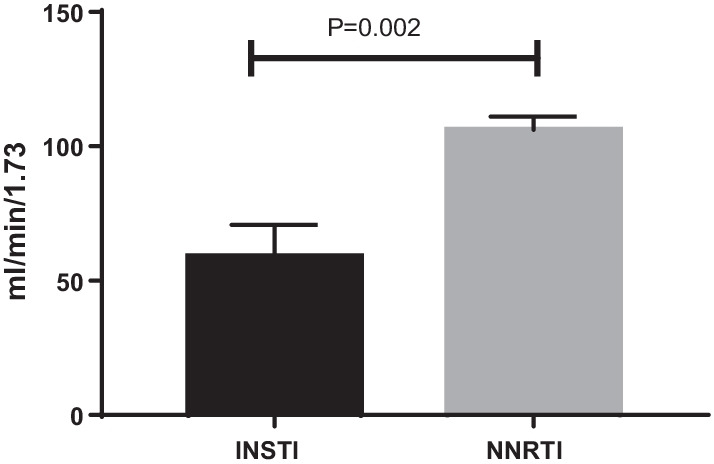
Glomerular filtration rate: as an index of kidney function, eGFR values showed normal levels in the NNRTI (*n* = 6) group compared to abnormally low levels in INSTI (*n* = 13) group, which maybe an indication of CKD. The results were statistically significant (*p* = 0.002)

EGFR levels (as shown in Fig. 6) demonstrate a statistically significant (*p* = 0.002) difference in glomerular filtration rate between the INSTI group (59 SEM + 8.04) and NNRTI group (106.16 + 4.94), with lower eGFR in INSTI group. The data suggest that in the INSTI group, there is a suggestion of early-stage chronic kidney disease (CKD).

#### Arterial stiffness

Stiffness of an artery significantly contributes to lack of pliability and contractility and is an important marker of increased peripheral resistance, diastolic dysfunction and systemic hypertension. The results of arterial stiffness measurements may indicate early stages of kidney damage. It is associated with cardiovascular diseases in older individuals and is positively associated with hypertension, coronary artery disease, stroke, heart failure and atrial fibrillation. Arterial stiffness is assessed using parameters such as AI adjusted for a heart rate of 75 (AI-75) and PWV.

Average augmentation index-75 is higher in the INSTI group (17.08 ± 3.97) as compared to NNRTI group (12.83 ± 4.85) but not statistically significant. Similar trend was observed with average augmentation pressure with higher values in INSTI group (9.08 ± 1.85) as compared to NNRTI group (7.83 ± 2.85).

## Discussion

This is a novel study where we compared two commonly used HIV medication regimens. Currently there are seven different classes of HIV medications, non-nucleoside reverse transcriptase inhibitors (NNRTIs), nucleoside reverse transcriptase inhibitors (NRTIs), protease inhibitors (PIs), fusion inhibitors, CCR5 antagonists, integrase strand transfer inhibitors (INSTIs) and post-attachment inhibitors, as eluded before.

An initial antiretroviral regimen generally consists of two nucleoside/nucleotide reverse transcriptase inhibitors (NRTIs) in combination with a third active drug from one of the following classes: non-nucleoside reverse transcriptase inhibitor (NNRTI), protease inhibitor (PI; boosted with ritonavir or cobicistat) or integrase strand transfer inhibitor (INSTI). PI-based regimens have been implicated in causing weight gain, dyslipidemia and worsening of insulin resistance [12]. Therefore, we embarked on trying to differentiate between NNRTI and INSTI as part of a medication regimen, which contained two NRTI at a baseline, frequently tenofovir–emtricitabine.

As far as the differences between the regimens, the interesting measures were mostly cell based with number of progenitor cells both CD133 and CD34 higher in INSTI group indicating better CVD risk profile, based on the published literature [14]. The effects of INSTI versus NNRTIs are consistent with the results from gene expression studies (Fig. 2) in which two antioxidants, SOD2 and Catalase mRNA expressions showing higher expressions in INSTI group in CD34+ cells with Catalase result showing statistical difference.

A bonafide cellular marker of inflammation such as Neutrophil–Lymphocyte Ratio (NLR) was also lower in INSTI group (Fig. 3).

As initial manifestations of metabolic dysfunction, INSTI group showed lower fasting glucose levels versus NNRTI (Fig. 4) indicating greater metabolic dysfunction in NNRTI group. Inflammatory marker levels such as interleukin 6 or IL-6 and hsCRP were very similar to each other in the two groups.

It is well known that increased albuminuria is an early sign of cardiovascular abnormality. In both diabetes and metabolic syndrome, one notes the presence of albuminuria ahead of overt expression of other vascular complications [20].

Though the difference failed to reach statistical significance (*p* = 0.08) the mean urine microalbumin was modestly higher in INSTI group compared to NNRTI group (Fig. 5). A lower GFR in INSTI group (Fig. 6) in the context of high levels of proteinuria is interesting. Higher levels of proteinuria indicate renal podocyte damage. Podocyte damage may be associated with impaired renal function, which is often associated with low eGFR. This may be a possibility with INSTI treatment. Arterial stiffness parameters [15] did not show statistical difference between the groups.

Considering the results of these analyses, the statistically significant differences such as progenitor cell number increase, increased gene expression of antioxidants and inflammatory markers genes in INSTI group along with NLR values, we conclude that INSTI medication group most likely provides better and sustained cardiometabolic risk reduction to HIV subjects. The lower fasting glucose levels were also reassuring in INSTI group.

While our non-cellular results (both plasma values and microalbuminuria) did not show a statistically significant difference, our cellular results, based on CD34+ cells actually, did show statistically significant differences in number and protective antioxidant gene expressions such as Catalase and SOD2. Our results once again demonstrate that the cellular results may be of more value even if the cohort size is small, compared to non-cellular results, though the latter is more commonly used clinically.

However certain well-established clinical parameters such as higher albuminuria, with poor eGFR along indicate some concern with INSTI group, impacting renal function.

### Limitations

It is to be acknowledged that this study was designed as preliminary study and the results need to be interpreted in that context. Only males were included to avoid gender as a confounder in a small population. The small sample size in the NNRTI group and consequent low statistical power, statistics for the 2 groups needs to be interpreted descriptively (Table 2) though we have calculated the *p* values. The most noteworthy differences were clearly cell-based assays; however, those differences may be due to chance and will need to be replicated in a larger study.

## Conclusions

We conclude that the INSTI-based HAART regimen may provide a better CVD risk profile compared to NNRTI-based HAART regimen, based on hematopoietic stem cell-based assays, and provides with statistically significant differences between the groups even in this small cohort. Certain clinical parameters, however, are of concern such as the increased albuminuria along with lower eGFR, noted in INSTI group. Because of the small size, these results would need replication in additional studies.

## Data Availability

All data pertaining to the study are freely available.

## Appendix

**Table:**

Inclusion criteria	1. Male only (as gender variation of progenitor cells along with effect of estrogen, may lead to difficulty in data interpretation), Age above 40, but less than 70 years, 2. Body mass index (BMI) between 25.0 and 39.9 (both inclusive) 3. eGFR ≥ 50 mL/min/1.73 m^2^ by MDRD 4. Subject taking one of the HAART regimens containing either INSTI or (NNRTI or NRTI alone based regimens) of HAART
Exclusion criteria	1. Uncontrolled hyperglycemia with random blood glucose > 200 mg/dL (> 13.3 mmol/L) or HbA1c ≥ 6.5 2. Liver disease with ALT, AST or ALP × 3 ULN 3. Subjects with HCV and HBV and detectable HCV RNA or HBV DNA 4. GFR < 50 mL/min/1.73 m^2^ by MDRD 5. Prior surgery with chronic malabsorption (e.g., bariatric) in prior 1 year 6. Clinically significant RBC disorders such as hemoglobinopathies 7. Chronic use of anti-inflammatory drugs for the last 3 months 8. On statin medications and their LDL is under control (≤ 70) 9. Use of consistent long-term steroid medication (oral, inhaled, injected) in last 1 month 10. Treatment with a strong cytochrome P450 3A4 (CYP34A) or P-gp inducer (i.e., Rifampin) 11. Active smokers, active wounds or recent surgery within 1 month 12. Untreated hyper/hypothyroidism 13. Implanted devices (e.g., Pacemaker) that may interact with Tanita scale 14. Any other clinical condition that would jeopardize patient safety while participating 15. Chronic or persistent alcohol or drug abuse 16. Hypertriglyceridemia above 500 mg/dl 17. Prisoners or subjects who are involuntarily incarcerated 18. Subjects who are compulsorily detained for treatment of either a psychiatric illness 19. Participation in another trial with an investigational drug within 30 days prior to consent

## Abbreviations

INSTI: Integrase inhibitor
NNRTI: Non-nucleotide reverse transcriptase inhibitors
AI: Augmentation index
AI-75: Augmentation index adjusted for a heart rate of 75
AP: Augmentation pressure
AS: Arterial stiffness
BCl-2: B-cell lymphoma 2
BMI: Body mass index
BUN: Blood urea nitrogen
CAT: Catalase
CDKN1A: Cyclin-dependent kinase inhibitor 1A
CD34: Progenitor marker
CFU: Colony forming units
CVD: Cardiovascular disease
CXCR4: C-X-C motif chemokine receptor 4
DBP: Diastolic blood pressure
DPP-4 Inhibitors: Dipeptidyl peptidase-4 inhibitors
EDN-1: Endothelin 1
eGFR: Estimated glomerular filtration rate
ELISA: Enzyme-linked immunosorbent assay
eNOS: Nitric oxide synthase 3 (endothelial cell)
EPCs: Endothelial progenitor cells
FFM: Fat-free mass
GAPDH: Glyceraldehyde-3-phosphate dehydrogenase
GLP1: Glucagon like peptide 1
GPX1: Glutathione peroxidase 1
HbA1C: Hemoglobin A1C, glycated hemoglobin test
IGF1: Insulin-like growth factor 1
IL-6: Interleukin 6
kg: Kilograms (weight)
lbs: Pounds (weight)
MNCs: Mononuclear cells
PECAM1: Platelet and endothelial cell adhesion molecule 1
PWA: Pulse wave analysis
PWV: Pulse wave velocity
qRT-PCR: Quantitative reverse transcriptase polymerase chain reaction
REE: Resting energy expenditure
SBP: Systolic blood pressure
SDF1α: Stromal cell-derived factor-1α
SOD1, SOD2: Super oxide dismutase 1 & 2
TBW: Total body water
TNFα: Tumor necrosis factor α
TP53: Tumor protein p53
VEGF: Vascular endothelial growth factor
VEGFA: Vascular endothelial growth factor A
VEGFR2: Vascular endothelial growth factor receptor 2
